# Hepatic venous pressure gradient after balloon-occluded retrograde transvenous obliteration and liver stiffness measurement predict the prognosis of patients with gastric varices

**DOI:** 10.1186/s12876-022-02616-z

**Published:** 2022-12-22

**Authors:** Yuki Shirane, Eisuke Murakami, Michio Imamura, Masanari Kosaka, Yusuke Johira, Ryoichi Miura, Serami Murakami, Shigeki Yano, Kei Amioka, Kensuke Naruto, Yuwa Ando, Shinsuke Uchikawa, Yuji Teraoka, Takuro Uchida, Hatsue Fujino, Atsushi Ono, Takashi Nakahara, Tomokazu Kawaoka, Daiki Miki, Masami Yamauchi, Wataru Okamoto, Masataka Tsuge, Keigo Chosa, Kazuo Awai, Hiroshi Aikata, Shiro Oka

**Affiliations:** 1 Department of Gastroenterology and Metabolism, Graduate School of Biomedical and Health Sciences, Hiroshima University, 1-2-3 Kasumi, Minami-Ku, Hiroshima, 734-8551 Japan; 2 Cancer Treatment Center, Hiroshima University Hospital, Hiroshima, Japan; 3 Natural Science Center for Basic Research and Development, Hiroshima University, Hiroshima, Japan; 4 Department of Diagnostic Radiology, Graduate School of Biomedical and Health Sciences, Hiroshima University, Hiroshima, Japan

**Keywords:** Balloon-occluded retrograde transvenous obliteration (BRTO), Esophageal varices, Gastric varices, Hepatic venous pressure gradient (HVPG), Liver stiffness measurement

## Abstract

**Background:**

Balloon-occluded retrograde transvenous obliteration (BRTO) is a treatment option for patients with gastric varices (GVs). This study aimed to clarify the clinical significance of portal hypertension estimated by the hepatic venous pressure gradient (HVPG), subsequent exacerbation of esophageal varices (EVs), and prognosis of patients who underwent BRTO for GVs.

**Methods:**

Thirty-six patients with GVs treated with BRTO were enrolled in this study, and their HVPG was measured before (pre-HVPG) and on the day after BRTO (post-HVPG). After BRTO, patients were followed-up for a median interval of 24.5 (3–140) months. Clinical factors related to EVs exacerbation and prognosis after BRTO were retrospectively analyzed.

**Results:**

Post-HVPG increased compared to pre-HVPG in 21 out of 36 patients (58%), and post-HVPG was overall significantly higher compared to pre-HVPG (*P* = 0.009). During the observation period, 19 patients (53%) developed EVs exacerbation, and the cumulative EVs exacerbation rates at 1, 3 and 5 years after BRTO were 27%, 67%, and 73%, respectively. Pre-HVPG was not related to EVs exacerbation, although elevation of post-HVPG to ≥ 13 mmHg (*P* < 0.01) and high level of serum aspartate aminotransferase (*P* < 0.05) were significant independent risk factors for EVs exacerbation after BRTO. Fourteen patients (38.9%) died during the observation period. An elevated value of liver stiffness measurement (LSM) of ≥ 21 kPa was a significant independent risk factor for poor prognosis after BRTO (*P* < 0.05).

**Conclusions:**

HVPG increases after BRTO. HVPG after BRTO has greater predictive ability for subsequent EVs exacerbation than HVPG before BRTO. LSM is a potential prognostic parameter in patients who undergo BRTO.

**Supplementary information:**

The online version contains supplementary material available at 10.1186/s12876-022-02616-z.

## Background

Gastric varices (GVs) are a complication of portal hypertension and are observed in approximately 20% of patients with liver cirrhosis [1, 2]. They are less prevalent than esophageal varices (EVs) and are present in 5–33% of patients with portal hypertension, with a reported incidence of bleeding of approximately 25% in 2 years, and a higher rate observed in patients with fundal varices [1, 3]. Although GVs bleeding is less frequent than EVs bleeding in patients with liver cirrhosis, bleeding from GVs is much more severe, requires more transfusion, and leads to a higher mortality rate [4, 5]. The treatment for GVs includes endoscopy, surgery, interventional radiology using a catheter, and conservative therapy [6–8]. The treatment of GVs bleeding also requires the involvement of a multidisciplinary team that includes hepatologists, endoscopists, diagnostic radiologists, and interventional radiologists [9].

Balloon-occluded retrograde transvenous obliteration (BRTO) is an interventional radiological treatment for GVs that was originally introduced in Japan [10–12]. BRTO is reported to be an effective strategy against GVs with a gastro-renal shunt, and reportedly results in a high GVs obliteration rate and low recurrence rate [13–18]. However, exacerbation of EVs is common (seen in 13–56% of cases after 1 year) after GVs eradication by BRTO, and several predictive factors are reported to be associated with the exacerbation of EVs [14, 16–20]. Hepatic venous pressure gradient (HVPG), which is calculated by measuring the difference between wedge hepatic venous pressure and free hepatic venous pressure, has been recognized as a reproducible and reliable method for assessing portal pressure and as a predictive factor for the development of EVs in patients with cirrhosis [3, 21–23]. However, few reports have described the clinical significance of HVPG in the prognosis of patients who have undergone a BRTO procedure and show EVs exacerbation [20, 23].

In the present study, the relationship between long-term prognosis, exacerbation of EVs, and portal hypertension estimated by HVPG was analyzed to identify predictive factors related to the prognosis of patients undergoing BRTO for GVs, and the incidence of EVs exacerbation in these patients.

## Methods

### Patients

**Fig. 1:**
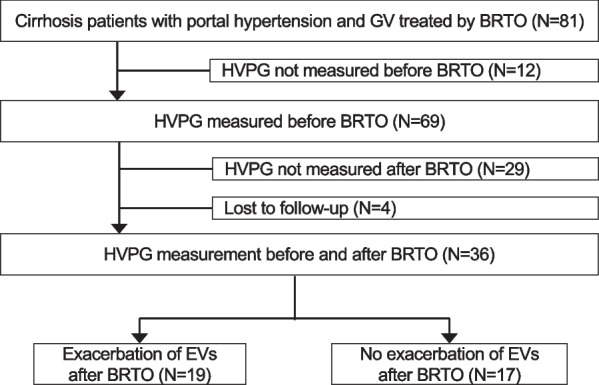
Flow diagram of the patients enrolled in the study

Figure 1 shows the flow diagram of the patients enrolled in the study. Eighty-one consecutive cirrhosis patients with portal hypertension who therapeutically or prophylactically underwent BRTO for GVs at Hiroshima University Hospital between January 2011 and December 2021 were initially included. Of the 81 patients, 12 and 29 patients were excluded due to a lack of measurement of HVPG before (pre-HVPG) and after BRTO (post-HVPG), respectively. Four patients were excluded due to loss to follow-up. As a result, 36 consecutive patients with both pre- and post-HVPG measurements were enrolled in this study. All patients underwent contrast-enhanced computed tomography (CT) scanning before BRTO to clarify the hemodynamics around the GVs, including the collateral vein and porto-systemic shunt [8]. Similarly, gastrointestinal endoscopic examinations were performed in all patients to confirm the indication of BRTO as GVs bleeding and/or GVs with rapid growth or appearance of a red color (RC) sign, or GVs classified as F3 based on the classification of the Japan Society for Portal Hypertension [24]. Patients in whom the drainage route from the GVs was not via the gastro-renal vein did not undergo BRTO [8].

This study was conducted in accordance with the ethical principles of the Declaration of Helsinki and approved by the Institutional Review Board of Hiroshima University. Written informed consent was obtained from each patient after providing them a detailed explanation of the study.

### BRTO procedure

BRTO was performed using the established procedure, as previously reported [14, 17]. Briefly, all patients underwent selective angiography of the celiac and superior mesenteric arteries, along with CT during arterial portography via the superior mesenteric arteries and/or splenic arteries using an angio-CT system (Aquilion LB, Canon Medical Systems, Ohtawara, Japan) to evaluate porto-systemic collaterals around the GVs via the femoral artery before BRTO. To perform the BRTO procedure, a 5-Fr balloon catheter (Selecon MP catheter; Terumo Clinical Supply, Gifu, Japan) was inserted into the gastro-renal shunt through the inferior vena cava via the right femoral or right jugular vein under local anesthesia. Balloon-occluded retrograde venography was performed to determine the hemodynamics of the GVs and collateral veins after blockade of outflow vessels from the gastro-renal shunt by balloon occlusion, to categorize the GVs according to Hirota’s classification [11]. After the GVs were visualized by retrograde venography under fluoroscopy, the BRTO procedure was performed using sclerosing agents, commonly 10% ethanolamine oleate (Oldamin; Takeda Pharmaceutical, Osaka, Japan) mixed with the same volume of non-ionic contrast medium iopamidol (Iopamiron 300; Bayer Healthcare, Osaka, Japan), with 5% ethanolamine oleate mixed with iopamidol (EOI) under balloon occlusion. When necessary, minor collateral shunt vessels were embolized using a 50% glucose solution or microcoils prior to the 5% EOI injection [25]. To prevent renal dysfunction related to hemolysis, which occurs as an adverse effect of 5% EOI, 2,000 or 4,000 units of haptoglobin was administered intravenously to all patients before BRTO. To avoid incomplete therapeutic efficacy and pulmonary infarction due to an unstable thrombus, the balloon catheter was retained in the draining vein with balloon inflation overnight, and removed after confirmation of complete obliteration of the varices by retrograde venography [25]. When obliteration of the shunt was insufficient on retrograde venography, an additional BRTO was subsequently performed until the inflow vessels disappeared. All patients underwent contrast-enhanced CT scanning and gastrointestinal endoscopy one week after BRTO to clarify the technical success, which was estimated by observation of low attenuation, disappearance of enhancement, and reduction in size of the GVs.

### HVPG measurement protocol

Prior to the BRTO procedure, baseline HVPG was measured using the 5-Fr balloon catheter, which was inserted through the superior or inferior vena cava into the right hepatic venous branch via the right femoral or right jugular vein [23, 26]. After setting the zero-reference point, free hepatic venous pressure and wedged hepatic venous pressure were measured using a nanometer (Polygraph MSC-7000; Fukuda Denshi, Tokyo, Japan) by taking enough time to stabilize the mean pressure. Pre-HVPG was calculated as the difference between the above-measured wedged and free venous pressures. On the day after BRTO, the retained balloon catheter in the draining shunt vein with balloon inflation overnight was removed after confirmation of complete obliteration by retrograde venography. Free hepatic venous pressure, wedged hepatic venous pressure, and HVPG were measured again on the day after BRTO (post-HVPG) before removing the balloon catheter system.

### Measurement of liver stiffness

Liver stiffness measurements were made by transient elastography based on ultrasound using a FibroScan-502® Touch system (Echosens, Paris, France) with M-probe and XL-probe. Patients were placed in the supine position with the right hand at maximal abduction for right lobe liver scanning. Measurements were considered valid when there were at least 10 measurements with LSM values of ≥ 60% and an interquartile range of < 30%, and the median value of these measurements was used for analysis.

### Follow-up after BRTO

Endoscopic findings of the EVs and GVs were evaluated according to the classification system of the Japanese Society for Portal Hypertension and Esophageal Varices [24]. The form (F) of EVs was classified as small straight (F1), enlarged tortuous (F2), or large coil-shaped (F3). The RC sign was also classified based on the criteria of the Japanese Society for Portal Hypertension and Esophageal Varices [24]. Endoscopic examinations were performed every 6 months or 1 year after the BRTO to follow-up the EVs and GVs. Worsening of the form and RC sign compared to baseline on follow-up endoscopy was defined as aggravation of EVs. The images were evaluated by two expert endoscopists. Aggravated EVs were treated by endoscopic injection sclerotherapy (EIS) or endoscopic variceal ligation (EVL) when the attending physicians determined that treatment for the EVs was needed.

### Statistical analysis

Laboratory and physical data were collected before BRTO, and survival and EVs aggravation were examined after BRTO, according to our previous report [17]. Quantitative variables were compared using the Wilcoxon signed-rank test. Optimal cut-off points for quantitative variables were determined by receiver operating characteristic curve analysis, if necessary. Cumulative survival and EVs aggravation rates were determined using the Kaplan–Meier method, and cumulative curves between subgroups were compared using the log-rank test. The Cox proportional hazard model was used to estimate the significance of the independent variables. Statistical significance was set at *P* < 0.05. Statistical analyses were performed using IBM SPSS Statistics for Windows (version 22.0; IBM Corp., Armonk, NY, USA).

## Results

### Clinical course of the patients who underwent BRTO

Baseline characteristics of the 36 patients who underwent BRTO are shown in Table 1. Of the 36 patients, seven patients (19%) underwent BRTO for GVs bleeding. The remaining 29 patients (81%) were prophylactically treated by BRTO because the GVs were diagnosed to have a high risk for bleeding based on endoscopic findings, such as presence of the RC sign or F3. The seven patients who experienced GVs bleeding underwent endoscopic or balloon tamponade treatment and subsequent BRTO. Clinical success of BRTO was achieved in all patients, as estimated by performing contrast-enhanced CT scans during the one week period following BRTO. None of the patients experienced severe complications related to BRTO treatment.

**Table 1:** Baseline characteristics of the enrolled patients

Characteristics	Values (N = 36)
Age (years)	71 (40–84)
Sex (male/female)	24/12
Etiology of cirrhosis	
Alcohol	9 [25.0%]
Hepatitis B virus	5 [13.9%]
Hepatitis C virus	13 [36.1%]
Others	9 [25.0%]
Laboratory data	
Albumin (g/dL)	3.5 (1.7–4.7)
Total bilirubin (mg/dL)	1.0 (0.4–4.0)
Prothrombin time (%)	81 (45–107)
Platelet count (× 10^3^/µL)	102 (56–402)
Aspartate aminotransferase (IU/L)	25 (18–133)
Alanine aminotransferase; (IU/L)	25 (4–81)
γ-Glutamyl transpeptidase (IU/L)	64 (12–351)
Ammonia (µg/dL)	55 (14–101)
Child–Pugh class (A/B/C)	22/12/2
Presence of splenomegaly	30 [83.3%]
Fib-4 index	4.79 (0.67–16.38)
Gastric variceal bleeding	7 [19.4%]
Co-existence of esophageal varices	23 [63.9%]
History of hepatocellular carcinoma	18 [50.0%]
Diameter of LGV before BRTO	4.5 (1–12)
Liver stiffness measurement (kPa)	30.2 (5.2–75.0)
Pre-HVPG (mmHg)	13 (1–25)
Post-HVPG (mmHg)	13 (5–25)

Continuous data are presented as the median and range, and categorical data are presented as the number of patients

LGV, left gastric vein; BRTO, balloon-occluded retrograde transvenous obliteration; HVPG, hepatic venous pressure gradient; Pre-HVPG, HVPG measured before BRTO; Post-HVPG, HVPG measured after BRTO

### HVPG measurement before and after BRTO

**Fig. 2:**
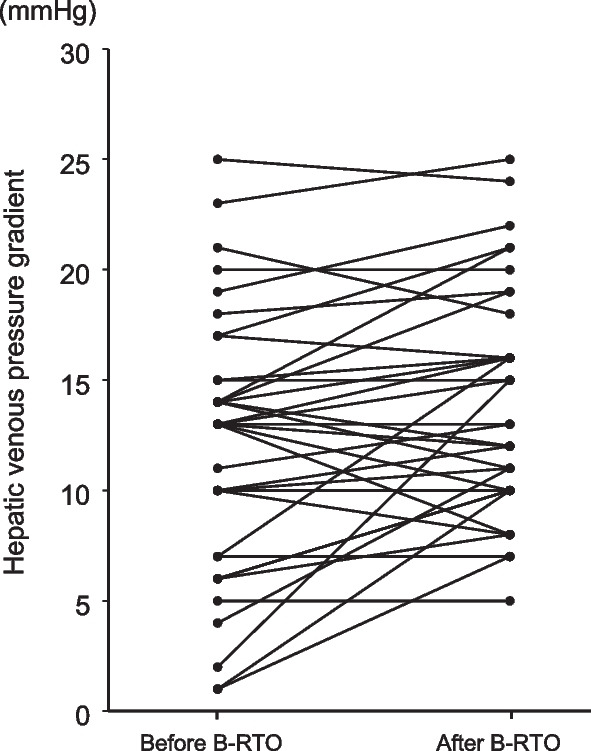
Changes in hepatic venous pressure gradients between before and after performing balloon-occluded retrograde transvenous obliteration (BRTO) in the 36 patients

Changes in HVPG in each patient are shown in Fig. 2. Before BRTO, the measured HVPG was higher than the normal limit (< 5 mmHg) [3], reflecting the presence of portal hypertension, in 31 out of 36 patients. After BRTO, HVPG increased in more than half of the patients. It increased in 21 patients (58.3%), was maintained in six patients (16.7%), and decreased in nine patients (25.0%). Post-HVPG was significantly elevated compared to the value of pre-HVPG (*P* < 0.01), and was higher than the normal limit in 35 out of 36 patients.

### EVs exacerbation after BRTO

**Fig. 3:**
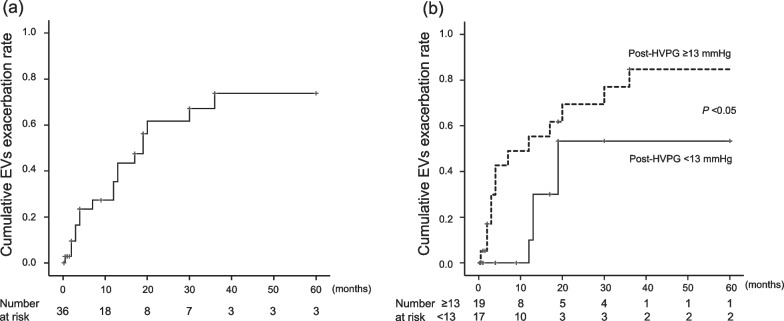
Cumulative esophageal varices (EVs) exacerbation rate after performing balloon-occluded retrograde transvenous obliteration (BRTO). **a** EVs were exacerbated in 19 patients (52.8%) who underwent BRTO therapy against gastric varices (GVs) after 10.5 months (median exacerbation time). **b** Cumulative EVs exacerbation rate according to hepatic venous pressure gradient (HVPG) measured after performing BRTO (post-HVPG)

After BRTO, patients were observed for a median duration of 24.5 (3–140) months. Twenty-three patients (63.9%) had co-existence of EVs before treatment, and EVs were exacerbated in 19 patients (52.8%) during the observation period. Among these patients, the exacerbated EVs were endoscopically treated in 12 patients: two patients by EIS and 10 patients by EVL. Cumulative EVs exacerbation rates 1, 3 and 5 years after BRTO were 27, 67 and 73%, respectively (Fig. 3a). Median time to the exacerbation of EVs was 10.5 months. Univariate analysis of the factors associated with EVs exacerbation after BRTO showed that aspartate aminotransferase (AST) and liver stiffness measurement (LSM) correlated significantly with EVs exacerbation (Table 2). Notably, post-HVPG also correlated with EVs exacerbation, although there was no correlation between pre-HVPG and EVs exacerbation. Multivariate analysis of the above factors identified AST ≥ 37 IU/L (hazard ratio [HR] 3.09, 95% CI 1.01–9.47, *P* = 0.04) and elevated post-HVPG of ≥ 13 mmHg (HR, 3.04, 95% CI 1.25–7.38, *P* = 0.01) as independent risk factors for EVs exacerbation after BRTO. Evaluation of optimal cut-off points of post-HVPG for predicting EVs exacerbation, as determined by receiver operating characteristic (ROC) curve analysis (Additional file 1: Fig. 1a), indicated an area under the curve (AUC) of 0.79 at the post-HVPG cut-off value ≥ 13 mmHg, with a sensitivity of 0.74 and specificity of 0.71. When the patients were classified by their post-HVPG values, patients with a post-HVPG value of ≥ 13 mmHg were more likely to develop EVs exacerbation compared to patients with post-HVPG values < 13 mmHg (Fig. 3b).

**Table 2:** Multivariate analysis of factors affecting exacerbation rate of esophageal varices after BRTO for gastric varices

Factors	Category	Univariate *P *value*	Multivariate	
			HR (95% CI)	*P *value**
Age (years)	< 71 vs. ≥ 71	0.22		
Sex	Male vs. Female	0.97		
Etiology	HBV plus HCV vs. others	0.74		
Albumin (g/dL)	< 3.5 vs. ≥ 3.5	0.15		
Total bilirubin (mg/dL)	< 1.0 vs. ≥ 1.0	0.16		
Prothrombin activity (%)	< 81 vs. ≥ 81	0.97		
Aspartate aminotransferase (IU/L)	< 37 vs. ≥ 37	0.04	3.09 (1.01–9.47)	0.04
Alanine aminotransferase (IU/L)	< 25 vs. ≥ 25	0.06		
γ-Glutamyl transpeptidase (IU/L)	< 64 vs. ≥ 64	0.10		
Platelet count (× 10^3^/µL)	< 102 vs. ≥ 102	0.74		
Ammonia (µg/dL)	< 55 vs. ≥ 55	0.05		
Child–Pugh class	A vs. B or C	0.26		
Fib-4 index	< 4.80 vs. ≥ 4.80	0.58		
Presence of splenomegaly	Yes vs. no	0.58		
Gastric varices bleeding	Yes vs. no	0.69		
Co-existence of esophageal varices	Yes vs. no	0.50		
History of hepatocellular carcinoma	Yes vs. no	0.88		
Diameter of LGV before BRTO	< 5 vs. ≥ 5	0.40		
Liver stiffness measurement (kPa)	< 30.2 vs. ≥ 30.2	0.01		
Pre-HVPG (mmHg)	< 13 vs. ≥ 13	0.06		
Post-HVPG (mmHg)	< 13 vs. ≥ 13	0.03	3.04 (1.25–7.38)	0.01

HBV, hepatitis B virus; HCV, hepatitis C virus; LGV, left gastric vein; BRTO, balloon-occluded retrograde transvenous obliteration; HVPG, hepatic venous pressure gradient; Pre-HVPG, HVPG measured before BRTO; Post-HVPG, HVPG measured after BRTO; HR, hazard ratio; CI, confidence interval

### Prognosis after BRTO

**Fig. 4:**
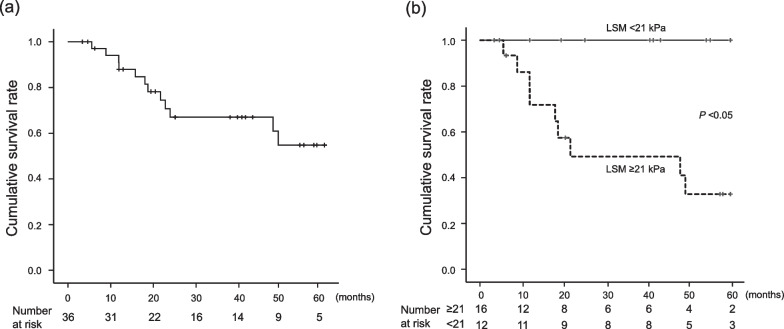
Cumulative survival rates after performing balloon-occluded retrograde transvenous obliteration (BRTO) for gastric varices. **a** Fourteen patients (38.9%) who underwent BRTO against gastric varices died after 24.5 months (median survival time). **b** Cumulative survival rate according to liver stiffness measurement (LSM)

During the observation period, 14 patients (38.9%) died due to various causes: six due to the progression of hepatocellular carcinoma (HCC), two due to infectious diseases, three due to gastroesophageal variceal bleeding, two due to liver failure, and one due to colon cancer. Cumulative survival rates 1, 3 and 5 years after BRTO were 88, 67 and 55%, respectively (Fig. 4a). In terms of factors associated with prognosis, univariate analysis showed that AST, γ-glutamyl transpeptidase and LSM had a significant correlation with poor prognosis after BRTO (Table 3). Multivariate analysis identified an LSM value of ≥ 25.1 kPa as an independent risk factor of poor prognosis after BRTO (HR, 8.27, 95% CI 1.04–65.52, *P* = 0.04). In addition, ROC curves, which were used to determine the cut-off value of LSM yielding the highest combined sensitivity and specificity with respect to prognosis after BRTO (Additional file 1: Fig. 1b), indicated a value of 25.1 kPa. ROC curve analysis at this cut-off LSM value showed an AUC of 0.78, sensitivity of 0.91 and specificity of 0.65. Interestingly, no patient with LSM < 25.1 kPa died during the observation period (Fig. 4b). In contrast, patients with LSM ≥ 25.1 kPa showed a significantly poor survival rate compared to patients with LSM < 25.1 kPa.

**Table 3:** Multivariate analysis of the clinical factors associated with cumulative survival after BRTO for gastric varices

Factors	Category	Univariate	Multivariate	
		*P *value*	HR (95% CI)	*P *value**
Age (years)	< 71 vs. ≥ 71	0.79		
Sex	Male vs. Female	0.38		
Etiology	HBV plus HCV vs. others	0.55		
Albumin (g/dL)	< 3.5 vs. ≥ 3.5	0.97		
Total bilirubin (mg/dL)	< 1.0 vs. ≥ 1.0	0.51		
Prothrombin activity (%)	< 81 vs. ≥ 81	0.14		
Aspartate aminotransferase (IU/L)	< 37 vs. ≥ 37	0.04		
Alanine aminotransferase (IU/L)	< 25 vs. ≥ 25	0.19		
γ-Glutamyl transpeptidase (IU/L)	< 64 vs. ≥ 64	0.02		
Platelet count (× 10^3^/µL)	< 102 vs. ≥ 102	0.68		
Ammonia (µg/dL)	< 55 vs. ≥ 55	0.05		
Child–Pugh class	A vs. B or C	0.32		
Fib-4 index	< 4.80 vs. ≥ 4.80	0.20		
Presence of splenomegaly	Yes vs. no	0.46		
Presence of large porto-systemic shunt	Yes vs. no	0.42		
Gastric varices bleeding	Yes vs. no	0.43		
Co-existence of esophageal varices	Yes vs. no	0.29		
History of hepatocellular carcinoma	Yes vs. no	0.07		
Diameter of LGV before BRTO	< 5 vs. ≥ 5	0.83		
Liver stiffness measurement (kPa)	< 25.1 vs. ≥ 25.1	0.02	8.27 (1.04–65.52)	0.04
Pre-HVPG (mmHg)	< 13 vs. ≥ 13	0.79		
Post-HVPG (mmHg)	< 13 vs. ≥ 13	0.05		

HBV, hepatitis B virus; HCV, hepatitis C virus; LGV, left gastric vein; BRTO, balloon-occluded retrograde transvenous obliteration; HVPG, hepatic venous pressure gradient; Pre-HVPG, HVPG measured before BRTO; Post-HVPG, HVPG measured after BRTO; HR, hazard ratio; CI, confidence interval

## Discussion

GVs are complications of portal hypertension and are less prevalent than EVs [1, 3]. However, bleeding from GVs is much more severe than that from EVs and leads to a higher mortality rate [4, 5]. Thus, treatment of GVs caused by portal hypertension is important when treating patients with liver cirrhosis. BRTO is an interventional radiological treatment for GVs with gastro-renal shunts that has been reported to provide a favorable GVs obliteration success rate (92.1–97.4%) [15–18]. On the other hand, exacerbation of EVs has been reported to occur after BRTO due to portal hypertension, with a cumulative exacerbation rate of 8.6–27% at 1 year, 20–58% at 3 years, and 18–66% at 5 years after BRTO [15–18]. Obstruction of a large gastro-renal shunt and the change in hemodynamics caused by BRTO appear to lead to the aggravation of EVs, although the mechanism of the exacerbation remains unclear [16]. Waguri et al. reported that concomitant partial splenic embolization (PSE) with BRTO might contribute to prevention of the exacerbation of EVs after BRTO [27]. In the present study, however, the phenomenon could not be analyzed because none of the cases underwent concomitant PSE with BRTO. In terms of portal vein thrombosis, three out of the 18 patients with HCC had portal vein tumor thrombosis, among whom only one patient had exacerbation of EVs after BRTO. Additionally, there was no statistically significant difference in the cumulative exacerbation of EVs with or without portal vein tumor thrombus. According to several reports, including our previous report, co-existence of EVs was a predictive factor for the exacerbation of EVs after BRTO [15–17]. In the present study, the co-existence of EVs was not related to the exacerbation of EVs after BRTO, despite the fact that the co-existence rates were similar to those observed in previous reports (63.9 vs. 45.6–68.0%) [15, 17].

HVPG has been widely used to assess portal pressure and the predictive value of EVs development in cirrhotic patients [3, 21, 22]. BRTO has been reported to significantly elevate HVPG [23, 28], as was also observed in the present study. Jogo et al. reported that an HVPG of ≥13 mmHg before BRTO is an independent risk factor for EVs aggravation after BRTO [20]. Additionally, there are some reports that elevation of HVPG after BRTO is related to the aggravation of EVs after BRTO [23, 29]. In the present study, post-HVPG correlated more significantly with EVs exacerbation after BRTO than pre-HVPG or the elevation of HVPG. Since obstruction of large gastro-renal shunt vessels is not inevitable during the BRTO procedure, post-HVPG might be a much more reliable parameter for monitoring of EVs exacerbation over an extended time period and might closely reflect portal hypertension and hemodynamics after BRTO.It has been reported that HVPG <12 mmHg and a reduction in HVPG by ≥20% of the baseline value reduces the risk of bleeding and death in cirrhotic patients with portal hypertension [30]. In the present study, as well, patients with post-HVPG ≥13 mmHg tended to have a poor prognosis. This suggests that a high portal pressure after BRTO might increase the risk of variceal or non-variceal complications in patients with GVs.

Left gastric vein (LGV) diameter and LSM have also been reported to be important risk factors for worsening of EVs after BRTO [31, 32]. The present study failed to show the correlation between LGV diameter and exacerbation of EVs, although the LSM value showed a correlation with EVs exacerbation after BRTO, with patients with high LSM values being more likely to develop exacerbation of EVs. LSM is useful for the non-invasive assessment of not only liver fibrosis, but also portal hypertension [33]. Both liver stiffness and splenic stiffness strongly correlate with HVPG in cirrhotic patients with large esophageal varices [34], and LSM positively correlates with the presence of EVs in patients with HCV-related cirrhosis [35]. Ogasawara et al. reported that LSM at 24 weeks after anti-HCV therapy is a predictor of EVs exacerbation after achieving SVR in patients with HCV-related cirrhosis [36]. In the future, studies involving a larger number of patients and more detailed analysis of the interrelation between HVPG, LGV diameter and liver and spleen stiffness are required to clarify the predictive factors and prevent the exacerbation of EVs after BRTO.

In the present study, patients with high LSM showed a poor prognosis after BRTO. Ishikawa et al. reported that LSM < 21.6 kPa predicted improvements in Model for End-stage Liver Disease (MELD) sodium scores and in survival rates after portosystemic shunt occlusion by BRTO [37]. The present study showed a similar cut-off value of LSM; patients with LSM < 21 kPa had a more favorable prognosis compared to those with LSM ≥ 21 kPa. As previously described, LSM seems to reflect both liver fibrosis and portal hypertension. Therefore, LSM might perceptively predict the exacerbation of EVs and prognosis after BRTO, and patients with high LSM levels seem to need careful consideration for possible subsequent exacerbation of EVs and poor prognosis after BRTO.

The present study has certain limitations. First, is the small sample size and the short observation periods. Although the study included only 36 patients, our results might provide basic clinical data for further large-scale cohort studies or validation studies aimed at identifying predictive factors of unfavorable outcomes in patients undergoing BRTO. Second, there might have been a selection bias. The present study included two patients with Child–Pugh class C cirrhosis. Whether or not BRTO should be introduced in patients with poor hepatic functional reserve, such as those with Child–Pugh class C cirrhosis, depends on the patient’s preference and clinician’s discretion. In addition, cases that underwent treatment for GVs bleeding were included, which might have affected the study results. Thus, further analysis is needed to clarify factors associated with the prognosis after BRTO using a larger number of patients, including those with poor hepatic functional reserve or GVs bleeding.

## Conclusions

In conclusion, HVPG, reflecting the degree of portal hypertension, increases after BRTO. HVPG after BRTO predicts subsequent EVs exacerbation more perceptively than HVPG before BRTO. LSM is a potential prognostic parameter in patients who have undergone BRTO.

## Supplementary information

**Additional file 1. Supplementary figure 1**. (a) Receiver operating characteristic (ROC) curve of hepatic venous pressure gradient (HVPG) measured after performing balloon-occluded retrograde transvenous obliteration (BRTO) (post-HVPG) and exacerbation of esophageal varices (EVs) after BRTO. (b) ROC curve of liver stiffness measurement (LSM) and prognosis after BRTO.

## Abbreviations

AST: Aspartate aminotransferase
AUC: Area under the curve
BRTO: Balloon-occluded retrograde transvenous obliteration
CT: Computed tomography
EIS: Endoscopic injection sclerotherapy
EVL: Endoscopic variceal ligation
EVs: Esophageal varices
GVs: Gastric varices
HCC: Hepatocellular carcinoma
HR: Hazard ratio
HVPG: Hepatic venous pressure gradient
LGV: Left gastric vein
LSM: Liver stiffness measurement
RC: Red color
ROC: Receiver operating characteristic

## Declarations

### Availability of data and materials

The datasets used and analyzed during the current study are available from the corresponding author on reasonable request.
