# Supplementary material for: Hepatic venous pressure gradient after balloon-occluded retrograde transvenous obliteration and liver stiffness measurement predict the prognosis of patients with gastric varices
Source: BMC Gastroenterol. 2022 Dec 22;22:535. doi: 10.1186/s12876-022-02616-z (PMC9773455; doi:10.1186/s12876-022-02616-z)
Supplement: Supplementary file 1 — Additional file 1. Supplementary figure 1. (a) Receiver operating characteristic (ROC) curve of hepatic venous pressure gradient (HVPG) measured after performing balloon-occluded retrograde transvenous obliteration (BRTO) (post-HVPG) and exacerbation of esophageal varices (EVs) after BRTO. (b) ROC curve of liver stiffness measurement (LSM) and prognosis after BRTO. [file 12876_2022_2616_MOESM1_ESM.pdf]

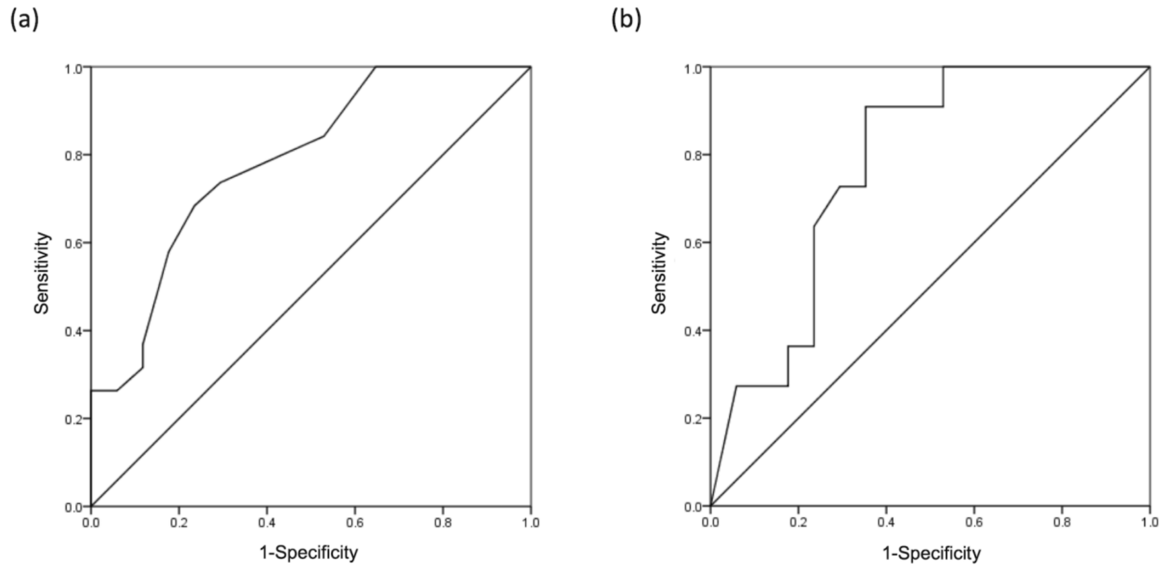

Supplementary figure 1.

(a) Receiver operating characteristic (ROC) curve of hepatic venous pressure gradient (HVPG) measured after performing balloon-occluded retrograde transvenous obliteration (BRTO) (post-HVPG) and exacerbation of esophageal varices (EVs) after BRTO. (b) ROC curve of liver stiffness measurement (LSM) and prognosis after BRTO.
